# mRNA vaccine platforms: linking infectious disease prevention and cancer immunotherapy

**DOI:** 10.3389/fbioe.2025.1547025

**Published:** 2025-03-12

**Authors:** Dariush Haghmorad, Majid Eslami, Niloufar Orooji, Iryna Halabitska, Iryna Kamyshna, Oleksandr Kamyshnyi, Valentyn Oksenych

**Affiliations:** ^1^ Department of Immunology, School of Medicine, Semnan University of Medical Sciences, Semnan, Iran; ^2^ Department of Bacteriology and Virology, Semnan University of Medical Sciences, Semnan, Iran; ^3^ Student Research Committee, Semnan University of Medical Sciences, Semnan, Iran; ^4^ Department of Therapy and Family Medicine, I. Horbachevsky Ternopil National Medical University, Ternopil, Ukraine; ^5^ Department of Medical Rehabilitation, I. Horbachevsky Ternopil National Medical University, Ternopil, Ukraine; ^6^ Department of Microbiology, Virology, and Immunology, I. Horbachevsky Ternopil National Medical University, Ternopil, Ukraine; ^7^ Department of Clinical and Molecular Medicine, Norwegian University of Science and Technology (NTNU), Trondheim, Norway

**Keywords:** mRNA vaccines, infectious diseases, cancer immunotherapy, vaccine development, vaccine delivery systems

## Abstract

The advent of mRNA vaccines, accelerated by the global response to the COVID-19 pandemic, marks a transformative shift in vaccine technology. In this article, we discuss the development, current applications, and prospects of mRNA vaccines for both the prevention and treatment of infectious diseases and oncology. By leveraging the capacity to encode antigens within host cells directly, mRNA vaccines provide a versatile and scalable platform suitable for addressing a broad spectrum of pathogens and tumor-specific antigens. We highlight recent advancements in mRNA vaccine design, innovative delivery mechanisms, and ongoing clinical trials, with particular emphasis on their efficacy in combating infectious diseases, such as COVID-19, Zika, and influenza, as well as their emerging potential in cancer immunotherapy. We also address critical challenges, including vaccine stability, optimization of immune responses, and the broader issue of global accessibility. Finally, we review potential strategies for advancing next-generation mRNA vaccines, with the aim of overcoming current limitations in vaccine technology and enhancing both preventive and therapeutic approaches for infectious and oncological diseases.

## 1 Introduction to mRNA vaccines: mechanisms and recent successes

Vaccination methods have evolved significantly, progressing from early approaches that utilized inactivated or attenuated pathogens to more sophisticated methods involving subunit vaccines composed of specific pathogen components that elicit targeted immune responses. Key advancements in vaccine research include recombinant viral vector vaccines, virus-like particle-based vaccines, conjugated polysaccharide or protein-based vaccines, and toxoid vaccines. A particularly notable achievement is the rapid development and approval of mRNA vaccines during the COVID-19 pandemic, which leverage mRNA technology to facilitate intracellular antigen production, marking a major milestone in vaccine science (Gote et al., 2023a).

Traditional vaccines, such as recombinant viral vector vaccines, protein-based vaccines, and DNA-based vaccines, have played significant roles in disease prevention. Recombinant viral vector vaccines use genetically engineered viruses to deliver specific antigens, triggering immune responses; however, their development is time-consuming, and they may pose safety concerns for immunocompromised individuals (Cid and Bolívar, 2021). Protein-based vaccines rely on purified proteins or protein fragments to elicit immunity, offering high safety but often requiring adjuvants to enhance their efficacy. DNA-based vaccines involve the delivery of DNA plasmids encoding antigens, which are cost-effective and stable but face challenges such as poor transfection efficiency and the risk of integration into the host genome (Lu et al., 2024). In contrast, mRNA vaccines leverage the host’s cellular machinery to produce antigens, offering faster and more flexible development. Additionally, they avoid genomic integration risks and elicit robust immune responses. These advantages make mRNA vaccines a promising alternative to traditional approaches, particularly in rapidly evolving disease scenarios (Barbier et al., 2022).

mRNA vaccines utilize the mRNA molecule not only as an immunogen but also as an adjuvant, taking advantage of its intrinsic immunostimulatory properties. Upon intramuscular injection, mRNA vaccines activate the adaptive immune system through several pathways: transfection of muscle and skin cells, interaction with resident immune cells at the injection site-such as dendritic cells, macrophages, and Langerhans cells—and subsequent migration to secondary lymphoid organs, including lymph nodes and the spleen, which initiates and enhances T and B cell responses (Kim et al., 2021). A comprehensive understanding of mRNA vaccines necessitates an examination of their physical mechanisms and chemical compositions, especially in comparison to traditional vaccine platforms. mRNA vaccines operate by delivering synthetic messenger RNA into host cells, instructing them to produce specific antigens that elicit an immune response. This approach leverages the body’s cellular machinery for antigen production, eliminating the need for cultivating pathogens or proteins externally. In contrast, traditional vaccines often rely on inactivated pathogens, live-attenuated organisms, or subunit proteins to stimulate immunity. These conventional methods may require extensive cultivation and purification processes (Bouazzaoui et al., 2021). The structural composition of mRNA vaccines includes a single-stranded mRNA molecule with a 5′cap, a poly (A) tail at the 3′end, and an open reading frame flanked by untranslated regions. The 5′cap is crucial for initiating translation by binding to eukaryotic initiation factors. To protect the mRNA and facilitate its entry into cells, it is encapsulated within lipid nanoparticles (LNPs). These LNPs are typically composed of ionizable lipids, cholesterol, phospholipids, and polyethylene glycol (PEG)-lipid conjugates, which collectively enhance stability and delivery efficiency (Bouazzaoui et al., 2021).

Traditional vaccines, depending on their type, may contain whole pathogens, protein subunits, or polysaccharides, often combined with adjuvants to boost immunogenicity. The production of these components can involve complex biological systems and purification steps. The utilization of synthetic mRNA in vaccines offers several advantages over traditional methods. The cell-free synthesis of mRNA allows for rapid and scalable production, which is particularly beneficial during pandemic responses. Additionally, mRNA vaccines can be designed to encode virtually any protein antigen, providing flexibility in targeting various pathogens. The use of LNPs not only protects the mRNA from degradation but also facilitates efficient delivery into host cells, enhancing the overall efficacy of the vaccine (Kis et al., 2022).

**FIGURE 1:**
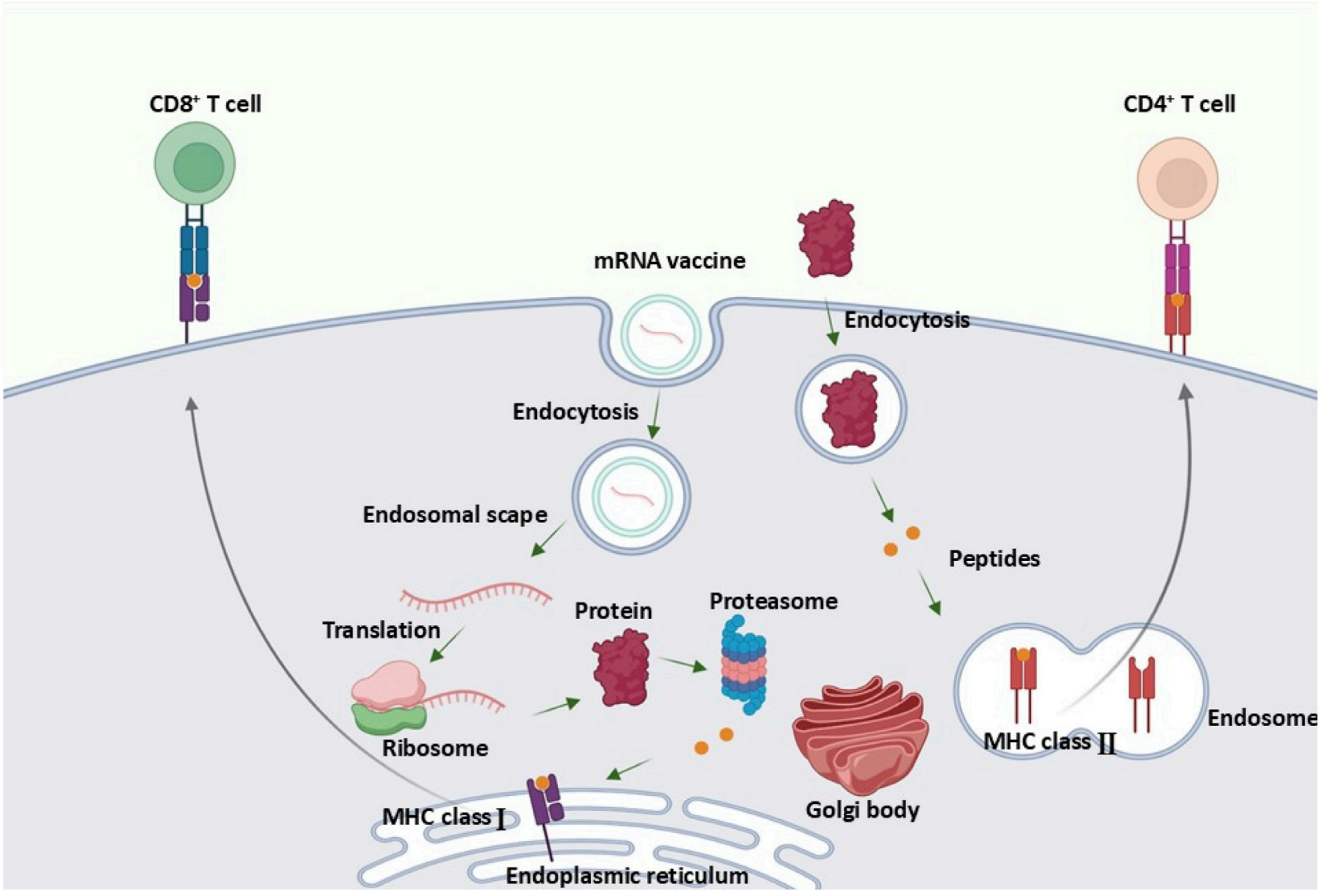
Mechanism of Action of mRNA Vaccines. mRNA vaccines stimulate antigen production, activating CD8^+^ and CD4^+^ T cells through MHC pathways and priming immune responses in lymph nodes. The Figure is designed using BioRender.com.

mRNA vaccines function by delivering mRNA into non-immune cells, prompting these cells to produce a specific target antigen. Following antigen production, proteasome-mediated processing reveals epitopes that bind to MHC class I molecules on antigen-presenting cells (APCs), such as CD8^+^ cytotoxic T cells, thereby enhancing cellular immunity against the antigen. Additionally, mRNA vaccines can stimulate bone marrow-derived dendritic cells (DCs) via the transfection of muscle cells, which further primes and activates CD8^+^ T cells for a robust immune response (Lazzaro et al., 2015). mRNA vaccines activate tissue-resident immune cells, including dendritic cells and macrophages, to initiate a localized immune response at the injection site (Alberer et al., 2017). mRNA transfection of immune cells prompts antigen presentation through MHC class I, leading to the maturation of CD8^+^ T cells. This process also stimulates APCs to utilize the MHC class II pathway, thereby activating CD4^+^ T helper cells (Figure 1) (Oberli et al., 2017). Following administration, the mRNA vaccine enters the lymphatic system and travels to the lymph nodes, which house monocytes as well as naïve T and B cells. This journey facilitates the transfection of APCs within the lymph nodes, subsequently leading to the priming and activation of both T and B cells (Firdessa-Fite and Creusot, 2020).

mRNA vaccines consist of large, negatively charged molecules that face challenges in penetrating cell membranes and maintaining stability in the bloodstream, as they are susceptible to degradation by nucleases and uptake by immune cells (Heine et al., 2021). To enhance mRNA delivery into cells, various techniques have been developed, including gene guns, electroporation, and *ex vivo* transfection. *In vivo* delivery methods often employ lipids or other transfection agents to facilitate the transfection of both immune and non-immune cells (Hajj and Whitehead, 2017).

Effective delivery of mRNA into cells is crucial for the success of mRNA vaccines. Two commonly employed techniques are electroporation and *ex vivo* transfection. Electroporation involves the application of a short burst of electrical pulses to create temporary pores in the cell membrane, allowing mRNA molecules to enter the cytoplasm. This method is widely used for dendritic cell-based cancer vaccines due to its efficiency in transfecting immune cells. However, the need for specialized equipment and cell damage caused by electrical pulses remain limitations (Dörrie et al., 2020). *Ex vivo* transfection, on the other hand, entails isolating cells (typically dendritic cells) from a patient, introducing mRNA into these cells in a laboratory setting, and then reintroducing the modified cells back into the patient. This approach ensures precise control over transfection conditions and is particularly advantageous for personalized cancer vaccines. However, it is labor-intensive, costly, and requires advanced clinical infrastructure (Han et al., 2019). In both methods, the goal is to achieve efficient mRNA entry into cells while preserving cell viability and enhancing antigen presentation for robust immune activation. Recent advancements, such as lipid nanoparticles (LNPs) for *in vivo* delivery, are also being explored to overcome the limitations of these traditional techniques (Jung et al., 2022).

Traditional vaccines often face significant limitations that can hinder their effectiveness in disease prevention and treatment. The development of dendritic cell vaccines is particularly labor-intensive and time-consuming, as it necessitates the generation of patient-autologous cells. Similarly, the creation of microorganism-based vaccines involves complex engineering and production processes. Furthermore, peptide vaccines tend to preferentially activate monoclonal T cells through MHC restriction, which can heighten the risk of immune escape (Matsui et al., 2018). DNA vaccines pose potential risks, including the possibility of genetic alterations, prolonged expression of the inserted DNA, and the development of anti-DNA autoantibodies. These factors may restrict their applicability in human populations (Fiedler et al., 2016; Wang et al., 2021). Selecting an effective vaccine format is essential for preventing and treating diseases. mRNA vaccines, which utilize a single nucleotide sequence as a template for protein translation, provide several advantages over traditional vaccination methods. Initially, these vaccines attracted limited investment due to concerns regarding their instability, suboptimal *in vivo* transfer, and potential innate immunogenicity. Nevertheless, ongoing research into mRNA has persisted, driven by its safety, straightforward design, and ease of manufacturing. This perseverance culminated in the development of highly effective COVID-19 mRNA vaccines, which have proven critical for efforts to control the pandemic (Table 1) (Zhang et al., 2023a).

**TABLE 1:** Comparative analysis of traditional vaccine limitations and the advantages of mRNA vaccines (Hsiung et al., 2024; Gupta et al., 2023).

Vaccine type	Limitations	How mRNA vaccines address these limitations
Inactivated/Live Attenuated Vaccines	Risk of reversion to virulent forms; complex production processes	No risk of reversion; streamlined and scalable production
Recombinant Viral Vector Vaccines	Potential for pre-existing immunity to vectors	Avoids the need for viral vectors by directly delivering synthetic mRNA
Protein-Based Vaccines	Requires adjuvants for strong immune response; time-consuming development	Strong immunogenicity without the need for adjuvants
DNA Vaccines	Risk of genomic integration; low transfection efficiency	No risk of genomic integration; high transfection efficiency
Peptide-Based Vaccines	Limited immune response due to MHC restriction	Broad immune activation through both MHC class I and II pathways

## 2 Dual applications of mRNA vaccines: infectious disease control and cancer immunotherapy

### 2.1 mRNA vaccines in infectious disease

mRNA vaccines confer protection against infectious diseases by encoding specific antigens associated with those diseases. Numerous preclinical and clinical trials have demonstrated the effectiveness of mRNA vaccines in inducing antiviral immunity against a range of infectious agents, including SARS-CoV-2, Zika, HIV, influenza virus, CMV, RSV, varicella-zoster virus, and rabies virus (Zhang et al., 2023a). As of June 18, 2021, there were 185 COVID-19 vaccine candidates in preclinical development, alongside an additional 102 candidates undergoing clinical trials, of which 19 were mRNA vaccines. The U.S. Food and Drug Administration (FDA) granted emergency authorization for the Pfizer-BioNTech vaccine BNT162b2 on December 11, 2020, marking it as the first mRNA product approved for human use. A week later, the Moderna vaccine (mRNA-1273) received approval for use in the United States. Both the Pfizer-BioNTech and Moderna vaccines have demonstrated high efficacy and safety; however, their requirement for cold-chain storage presents logistical challenges. Specifically, mRNA-1273 can be stored at 4–8°C for up to 1 month and for 12 h at room temperature, while BNT162b2 requires storage at −60°C (Chaudhary et al., 2021).

In 2013, a self-amplifying mRNA vaccine utilizing lipid nanoparticles (LNP) based on DLinDMA was developed within 8 days in response to the H7N9 epidemic in China. However, the absence of Good Manufacturing Practice (GMP) procedures for mRNA production hindered advancement to a phase I clinical trial (Hekele et al., 2013). Efforts have also been made to develop a universal influenza vaccine that would eliminate the need for annual adjustments. Such a vaccine could confer immunity against multiple influenza strains and subtypes. In 2012, a study involved administering three intradermal injections of 80 µg of RNActive-mRNA encoding the hemagglutinin from the PR8 H1N1 strain to mice. This approach resulted in both homologous and heterologous immunity against H1N1 and H5N1 strains, respectively, and the mice demonstrated protection against a lethal viral dose (10× LD50) (Petsch et al., 2012). Since that time, mRNA-based influenza vaccines have been explored using a range of delivery methods, including DLinDMA, DOTAP, polyethylenimine, and cationic nanoemulsions. Additionally, various mRNA technologies, such as nucleoside-modified mRNAs and self-amplifying mRNAs, have been investigated, alongside alternative antigen targets (Brazzoli et al., 2016; Bahl et al., 2017; Freyn et al., 2020).

Recent research has identified the non-mutable stalk region of hemagglutinin as a promising target for universal vaccination against influenza (Nachbagauer et al., 2017). A primer-booster regimen involving a lipid nanoparticle (LNP)-based mRNA vaccine (30 µg) targeting the conserved hemagglutinin stalk from the Cal09 H1N1 strain elicited a strong stalk-specific antibody response in mice, ferrets, and rabbits. These broadly protective antibodies provided mice with homologous immunity against Cal09 H1N1, heterologous immunity against PR8 H1N1, and heterosubtypic immunity against H5N1, ultimately safeguarding them from lethal viral challenges (Pardi et al., 2018a). Furthermore, the conserved stalk region of hemagglutinin, known for its resistance to mutations, has recently been recognized as a promising target for the development of a universal influenza vaccine (Nachbagauer et al., 2017). Mice, ferrets, and rabbits exhibited a specific antibody response following a primer-booster regimen that utilized a 30 µg lipid nanoparticle (LNP)-based mRNA vaccine targeting the conserved hemagglutinin stalk from the Cal09 H1N1 strain. Ultimately, this vaccination conferred protection to the mice against a lethal viral challenge by generating broadly protective antibodies that provided immunity against the Cal09 H1N1 strain, along with heterologous immunity against PR8 H1N1 and heterosubtypic immunity against H5N1 (Pardi et al., 2018a). In 2016, Moderna evaluated two influenza vaccine candidates in separate phase I trials. These trials involved administering two intramuscular injections of LNPs encapsulating nucleoside-modified mRNA that encoded the complete hemagglutinin proteins from the H10N8 and H7N9 strains (Bahl et al., 2017).

Because a single serotype is responsible for all Zika infections, it is anticipated that a vaccine targeting an antigen from any Zika strain could confer protection against all strains of the virus (Dowd et al., 2016). Antibodies that neutralize the premembrane and envelope (prM-E) proteins can inhibit viral fusion, making prM-E an effective antigen candidate for mRNA vaccines against Zika virus. Research has shown that a single dose of 30 µg or 50 µg of LNP-encapsulated nucleoside-modified prM-E mRNA successfully protected mice and rhesus macaques from Zika infection (Pardi et al., 2017a). The envelope proteins of dengue and Zika viruses share 54%–59% sequence identity, raising the potential for cross-reactivity between dengue and Zika vaccine antibodies, which could exacerbate dengue symptoms in subsequent infections. To address this, Moderna and Washington University developed a modified prM-E mRNA vaccine with a mutated epitope to minimize cross-reactive effects. Mice that received two doses spaced 21 days apart showed effective protection against Zika and exhibited reduced levels of dengue-enhancing antibodies (Dejnirattisai et al., 2016; Richner et al., 2017). A study utilized a squalene-based nanocarrier to deliver mRNA encoding ZIKV-117 neutralizing monoclonal antibodies using a passive delivery approach. Immunocompromised mice were effectively protected from lethal viral infection with a single 40 µg dose administered either 1 day before or 1 day after viral inoculation (Erasmus et al., 2020).

Currently, there are 38 million individuals living with HIV worldwide, and this figure is projected to increase to 42 million by 2030 (Dybul et al., 2021). Despite over 30 years of research, an effective HIV vaccine has yet to be developed, largely due to the high antigenic diversity of the HIV envelope protein and the presence of an extensive glycan shield that conceals key epitopes (Mascola, 2015). Numerous preclinical studies have explored various delivery vehicles—including cationic nanoemulsions, DOTAP/DOPE liposomes, polymers, and ionizable LNPs—to administer mRNA vaccines encoding HIV proteins. However, outcomes have been highly variable. These findings indicate that novel antigens and optimized delivery mechanisms are essential to effectively target HIV (Gómez-Aguado et al., 2020). In one experiment, a 0.7 mg/kg intravenous injection of LNP-encapsulated, nucleoside-modified mRNA encoding the VRC01 antibody achieved antibody concentrations similar to those produced by 10–20 mg/kg doses of monoclonal antibody (Pardi et al., 2017b). Additionally, an mRNA-based HIV vaccine elicited both humoral and cellular immune responses in mice and primates. After priming and booster vaccinations, rhesus monkeys exhibited a 79% reduction in susceptibility to SHIV exposure (Zhang et al., 2021). The promise of mRNA vaccines for HIV prevention was highlighted in a recent phase I clinical trial of the mRNA vaccine eOD-GT8 60mer, which demonstrated a high safety profile and successfully induced broadly neutralizing antibodies in 97% of participants (35 out of 36) (Leggat et al., 2022).

Despite the significant burden posed by RSV and over 40 years of vaccine development efforts, an approved RSV vaccine has yet to be realized due to several challenges (HW et al., 1969). Current RSV vaccine candidates aim to prevent viral fusion by targeting the highly conserved F protein. Fortunately, mRNA vaccines can be engineered to encode stable structures of the F protein (Espeseth et al., 2020). Moderna is currently assessing three single-dose vaccine candidates targeting the prefusion F protein: mRNA-1172 and mRNA-1777 for adults, and mRNA-1345 for children. Phase I trials have demonstrated that mRNA-1777 elicits a robust immune response without causing serious adverse effects. Additionally, mRNA-1345 has been optimized to generate significantly higher antibody levels compared to mRNA-1777. The company plans to combine mRNA-1345 with another vaccine candidate to provide children with protection against multiple pathogens in a single inoculation (Aliprantis et al., 2021).

mRNA vaccines targeting the Ebola virus (EBOV) may offer a safer alternative to traditional virus-based vaccines, as they do not replicate within the body. In mouse models, an mRNA vaccine containing self-amplifying mRNA encoding the EBOV glycoprotein, delivered via a dendrimer nanoparticle, proved effective. This approach resulted in the production of glycoprotein-specific IgG antibodies and robust IFN-γ and IL-2 responses from CD8^+^ and CD4^+^ T cells. Both a two-dose regimen of 4 μg and a single 40 μg dose provided protection against a lethal viral challenge (Chahal et al., 2016). In a separate study, guinea pigs were administered two doses of 20 μg each of LNP-encapsulated, nucleoside-modified mRNA targeting the EBOV glycoprotein. This regimen resulted in elevated antibody levels and conferred protection against a lethal viral challenge (Meyer et al., 2018).

CureVac employed its RNActive platform to deliver unmodified mRNA encoding the rabies virus glycoprotein in its rabies vaccine candidate, CV7201. In preclinical trials, administration of two doses of 80 µg each, spaced 21 days apart, resulted in the production of high levels of neutralizing antibodies and stimulated antigen-specific CD4^+^ and CD8^+^ T cell responses in both mice and pigs (Schnee et al., 2016). Additionally, CureVac utilized proprietary LNPs from Acuitas Therapeutics to deliver their updated rabies vaccine candidate, CV7202. In preclinical studies, CV7202, which contains unmodified mRNA for the rabies virus glycoprotein, elicited robust antibody responses and stimulated CD8^+^ and CD4^+^ T cell activity. In non-human primates, administration of two doses of 100 µg each, spaced 28 days apart, was well tolerated and resulted in antibody levels that were 20 times higher than those produced by a licensed rabies vaccine (Lutz et al., 2017). Phase I results indicate that two doses of 1 µg each produce high neutralizing titers, eliciting significant adaptive immune responses while demonstrating excellent tolerability (Aldrich et al., 2021).

Macrophage migrating inhibitory factor (PMIF), a cytokine released by Plasmodium, impairs the development of long-term memory in T cells (Sun et al., 2012). Building on this discovery, a vaccine was developed utilizing a squalene-based cationic nanoemulsion that contains self-amplifying mRNA encoding PMIF. Administration of two doses of 15 µg enhanced the development of helper T cells and induced anti-Plasmodium IgG antibodies, as well as memory T cell responses. Furthermore, transferring T cells from vaccinated mice provided protection to unvaccinated mice against Plasmodium sporozoites (Baeza Garcia et al., 2018).

### 2.2 mRNA vaccines for cancer

The goal of mRNA-based vaccines is to stimulate or enhance an effective immune response against tumors. This is accomplished by utilizing synthetic mRNA that encodes tumor-associated or specific antigens. The mRNA can be delivered via autologous dendritic cells or through direct injections. Once inside the body, the mRNA is taken up by APCs, where it is processed and presented on MHC class I and II molecules. This processing activates CD8^+^ T cells and CD4^+^ T cells, the latter of which can further assist in activating B cells (Lorentzen et al., 2022). TriMix, an immunostimulant mRNA vaccine that expresses CD70, CD40L, and an active form of TLR4, has been shown to induce robust CD8^+^ T cell responses in patients with stage III or IV melanoma. This resulted in promising tumor response rates observed in a phase II clinical trial (Wilgenhof et al., 2013). Moderna is currently conducting a clinical trial (NCT03739931) for mRNA-252, an immunostimulant vaccine that encodes human OX40L, IL-23, and IL-36 for the treatment of lymphoma. Additionally, the BNT111 mRNA vaccine, which encodes four tumor-associated antigens (NY-ESO-1, MAGE-A3, tyrosinase, and TPTE), has demonstrated effectiveness in treating patients with melanoma (Sahin et al., 2020). DCs are recognized as the most effective APCs within the immune system and can serve as a vaccine platform by transfecting them with mRNA encoding tumor antigens. This can be accomplished using tumor-associated antigen (TAA) mRNA or total tumor RNA, each of which has its own set of advantages and disadvantages. Although DCs have the ability to internalize naked mRNA, electroporation is often employed to enhance transfection efficiency without the use of carriers. Following transfection, the DCs are reinfused to stimulate an immune response, and the addition of mRNAs encoding costimulatory molecules can further augment their immunostimulatory properties. However, this *ex vivo* loading approach is both costly and labor-intensive. An example of this strategy is a phase I trial utilizing autologous Langerhans-type DCs that express xenogeneic TRP-2 mRNA (Vishweshwaraiah and Dokholyan, 2022a).

**FIGURE 2:**
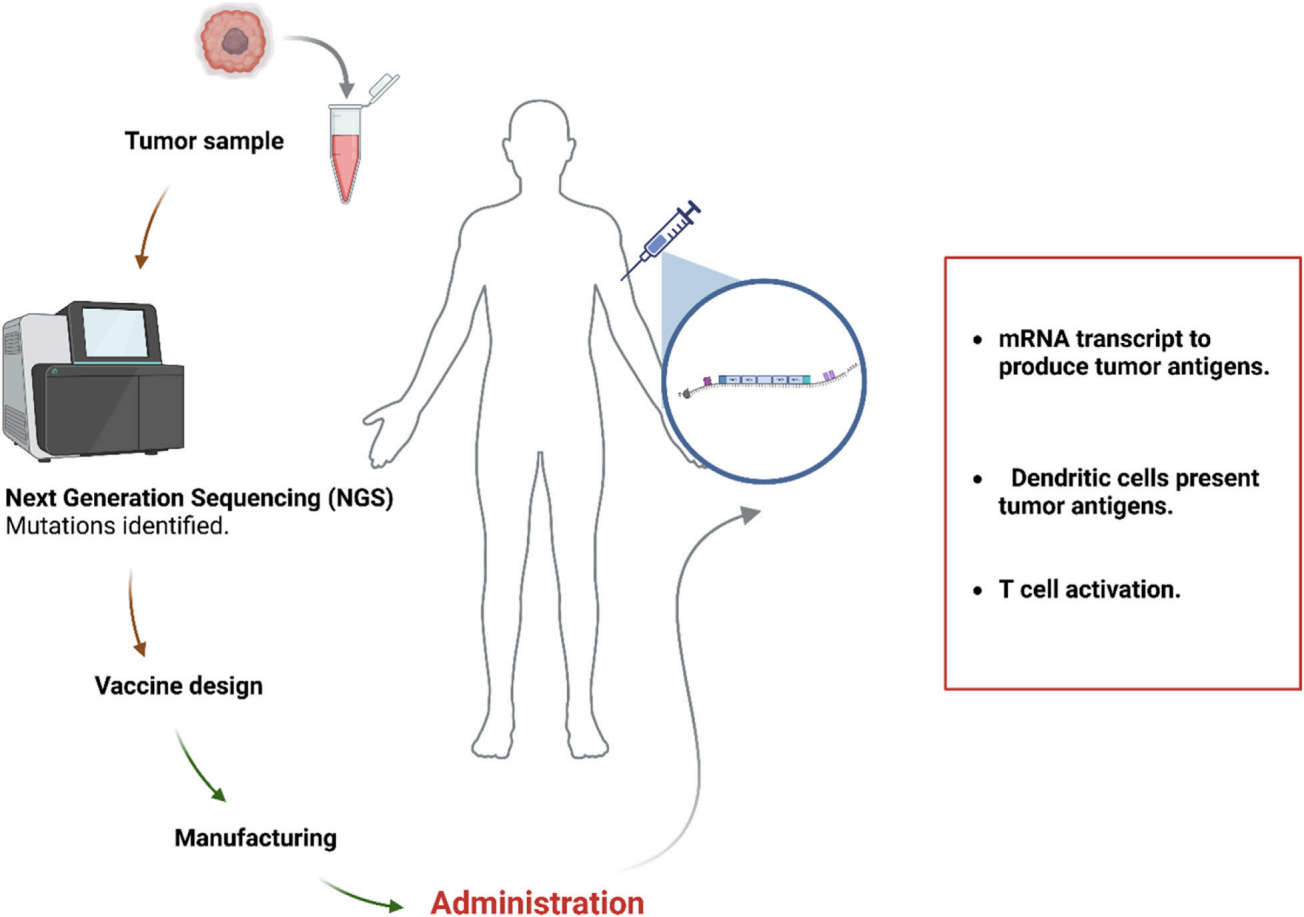
The process of developing personalized mRNA vaccines. The Figure is designed using BioRender.com.

In naked or unformulated mRNA vaccines, the mRNA molecules are present in a buffer solution (Sahin et al., 2017). APCs can directly receive antigens at the site of T-cell activation by administering non-formulated mRNA intranodally, thereby eliminating the necessity for APCs to migrate (Diken et al., 2011). Numerous studies have shown that dendritic cells are capable of absorbing non-formulated mRNA injected intranodally, which elicits robust anti-tumor T-cell responses (Sahin et al., 2017; Pardi et al., 2018b). Development of personalized mRNA vaccines requires recognizing and choosing neoantigens employing advanced sequencing and bioinformatics techniques. Next-generation sequencing (NGS), high-throughput screening, and machine learning algorithms have improved tumor-specific antigen (TSA) prediction by detecting mutations and unusual transcription/translation events in the exons of individual tumor genomes (Xu et al., 2021; Bassani-Sternberg et al., 2017; Kim et al., 2018; Figure 2).

Advancements in mRNA vaccine design have been significantly propelled by integrating next-generation sequencing (NGS), high-throughput screening, and machine learning algorithms. NGS technologies, such as VAX-seq, enable detailed profiling of vaccine-induced immune responses by analyzing the genetic composition of mRNA vaccines and their translation products. This comprehensive data collection facilitates the identification of specific mRNA sequences that optimize immune activation (Dash, 2024). High-throughput screening methods complement NGS by allowing rapid evaluation of extensive mRNA sequence libraries. For instance, screening approximately 12,000 synthetic 5′untranslated regions (5′UTRs) has led to the discovery of elements that enhance protein expression, crucial for effective vaccine development (Blanco et al., 2020). Machine learning algorithms further enhance this process by analyzing large datasets to predict which mRNA sequences and structures will yield optimal protein expression and immune responses. By learning from experimental data, these algorithms can guide the design of mRNA vaccines with improved efficacy (Imani et al., 2025). Collectively, the integration of NGS, high-throughput screening, and machine learning accelerates the development of mRNA vaccines by enabling precise design and optimization of vaccine components, leading to more effective immunizations.

In a phase 1 clinical trial, 13 patients with stage III or IV melanoma underwent intranodal administration of non-formulated mRNA vaccines designed to target individualized tumor mutation signatures. Each vaccine comprised ten selected neoepitopes specific to each patient. All participants demonstrated T-cell responses to multiple neoepitopes, and 40% of the five patients with stage IV melanoma exhibited clinical responses related to the vaccine (Sahin et al., 2017). mRNA-4157 and BNT122 are prominent personalized mRNA vaccines currently undergoing clinical trials. mRNA-4157 targets 34 specific mutations present in patients’ tumor DNA and is being investigated for melanoma, non-small cell lung cancer, and other solid tumors. A trial that combined mRNA-4157 with pembrolizumab in patients with head and neck squamous cell carcinoma reported a 50% overall response rate and a 90% disease control rate. Furthermore, two Phase IIb trials combining these treatments for high-risk melanoma patients indicated a 44% and 49% reduction in the risk of recurrence or death, respectively, suggesting enhanced benefits with prolonged treatment. This combination has advanced to Phase III trials (NCT05933577), marking it as the first mRNA cancer vaccine to reach this advanced stage (Wang B. et al., 2023).

Protamine-formulated mRNA vaccines, referred to as RNActive vaccines, utilize nucleotide-modified mRNA molecules combined with protamine. This combination is designed to enhance protein expression and increase immunogenicity. RNActive vaccines have been evaluated in various clinical trials (Lorentzen et al., 2022). In a placebo-controlled phase 1/2 study, patients with metastatic castration-resistant prostate cancer received an RNActive vaccine containing six prostate cancer-specific antigens (CV9104). While the vaccine was found to be safe for the patients, it did not demonstrate an improvement in overall survival or progression-free survival when compared to the placebo (Stenzl et al., 2017). In a phase 1/2 dose-escalation trial of the RNActive technology, patients with non-small-cell lung cancer (NSCLC) received the protamine-formulated mRNA vaccine CV9201, which targets five tumor-associated antigens. The trial included 46 patients, and while the vaccine was well tolerated and induced T-cell responses in 63% of evaluable patients, it did not enhance overall survival compared to historical controls (Sebastian et al., 2019).

mRNA-based cancer vaccines targeting viral antigens are garnering increasing attention, especially for cancers associated with viruses such as HPV, HBV, EBV, and HIV. A recent study demonstrated that an mRNA-HPV therapeutic vaccine (mHTV) exhibited robust immunogenicity and anti-tumor effects in both mice and non-human primates, underscoring its potential as both a therapeutic and preventive strategy for HPV-related cancers (Yao et al., 2024a).

Innovative strategies are being explored that involve delivering mRNA encoding the tumor suppressor p53 to liver cancer cells using nanoparticles, in combination with anti-PD-1 monoclonal antibodies. This approach has demonstrated effectiveness in modifying the tumor microenvironment and inducing anti-tumor responses (Xiao et al., 2022). Additionally, previous studies have employed mRNA encoding caTLR4 in conjunction with co-stimulatory ligands CD70 and CD40L to stimulate immune responses in APCs. This enhancement of T cell function has demonstrated efficacy in effectively targeting tumors and inhibiting early resectable breast cancer lesions in mice models (Van Lint et al., 2016). Phase I and Phase II clinical trials are currently underway to assess the safety and tolerability of mRNA-2752, an mRNA vaccine encoding three immune regulatory factors. This vaccine is being evaluated in patients with advanced cancers, both as a standalone treatment and in combination with a fixed dose of durvalumab, an anti-PD-L1 antibody developed by AstraZeneca (Bassani-Sternberg et al., 2017).

## 3 Advancements in mRNA delivery systems technology

mRNA vaccines are at the forefront of a medical revolution, offering a transformative approach to disease prevention and treatment. Unlike traditional vaccines, which rely on introducing weakened or inactivated pathogens to elicit an immune response, mRNA vaccines use the body’s cells as factories to produce specific proteins, known as antigens that trigger immunity. This groundbreaking method not only simplifies vaccine production but also accelerates the timeline for development, making it highly effective in responding to emerging or mutating infectious diseases, as demonstrated during the epidemic and pandemic disease (Baptista et al., 2021; Barberio et al., 2023). The safety profile of mRNA vaccines is one of its main benefits. Since mRNA vaccinations do not integrate into the host’s genome like DNA vaccines do, there is no chance of genomic change. Additionally, mRNA vaccines are particularly effective in inducing significant immune responses, both humoral and cellular, which offers substantial protection. mRNA vaccines may be produced in a matter of hours utilizing *in vitro* transcription methods, and the procedure does not include the use of viruses, living cells, or materials obtained from animals. Furthermore, by simply changing the encoded protein sequence, the modular structure of mRNA enables quick adaptation to other infections or variations, giving these vaccines great flexibility and the ability to quickly react to viral alterations (Linares-Fernández et al., 2020).

The applications of mRNA vaccines extend far beyond infectious diseases. They are also showing great promise in cancer immunotherapy. Therapeutic mRNA vaccines can instruct the immune system to target tumor antigens, potentially controlling tumor growth, eliminating residual cancer cells, and establishing long-term immune memory to prevent recurrence. This technology is being explored in various types of cancer, including melanoma, lung cancer, and cervical cancer (Barbier et al., 2022). Additionally, the potential of mRNA extends to other areas, such as treating genetic disorders by enabling the production of therapeutic proteins within the body, offering hope for patients with rare and chronic conditions. However, there are significant challenges associated with mRNA vaccine development and application (Rohner et al., 2022). One of the primary obstacles is the inherent instability of mRNA molecules, which are prone to degradation by enzymes in the body. This necessitates the use of advanced delivery systems like lipid nanoparticles (LNPs), which encapsulate the mRNA and protect it until it reaches its target cells. While LNPs have revolutionized mRNA delivery, making clinical applications possible, issues such as high production costs and the need for ultra-cold storage remain barriers to broader use (Makhijani et al., 2024a). Additionally, mRNA vaccines are associated with side effects, including fever, headaches, and, in some cases, more severe immune reactions. There have also been reports of autoimmune responses linked to the spike proteins used in COVID-19 mRNA vaccines, although these occurrences are relatively rare. A major issue is the inherent instability and immunogenicity of mRNA. Due to its large size and vulnerability to enzymatic degradation, mRNA requires protection through encapsulation, particularly from extracellular ribonucleases. While mRNA’s immunostimulatory properties can enhance immune responses, particularly for vaccines, excessive immune activation can diminish its effectiveness in other therapeutic applications. Efficient intracellular delivery is crucial, as mRNA must cross the cell membrane to reach the cytoplasm, where it can be translated into proteins. However, the negatively charged cell membrane poses a significant barrier due to electrostatic repulsion between the membrane and the similarly charged mRNA molecules (Okay et al., 2020).

In the future, the area of mRNA vaccines is expected to continue developing quickly. Personalized mRNA vaccines targeting tumor-specific antigens are shown significant promise in cancer therapy. Clinical trials are now investigating their ability to extend the duration of relapse-free survival. In addition to cancer, mRNA technology is being used to treat genetic illnesses such as metabolic diseases, where it may repair damaged proteins. The effectiveness and safety of mRNA-based therapeutics are being enhanced by advancements in delivery methods, especially ionizable lipids in LNPs. However, obstacles including extrahepatic targeting and large-scale manufacturing still need to be addressed (Gao et al., 2024). Recent innovations in delivery systems have focused on non-viral vectors, including lipid nanoparticles (LNPs), ionizable lipids, polymers, and dendrimers. LNPs have emerged as a leading method for encapsulating and delivering mRNA. These nanoparticles typically contain ionizable or cationic lipids that assist in overcoming cellular barriers and facilitate mRNA entry into the cytoplasm (Wahane et al., 2020). Polymers such as polyethyleneimine (PEI) and cationic nanoemulsions have also been explored to enhance stability and target specific tissues.

Effective delivery of mRNA is essential for vaccine development, with cationic carriers like PEI and cationic nanoemulsions showing great promise. PEI, a widely used cationic polymer, forms complexes with mRNA, protecting it from degradation and enhancing cellular uptake through the “proton sponge” effect, though its high cytotoxicity poses challenges. Researchers are modifying its structure to improve biocompatibility (Wu et al., 2024). Similarly, cationic nanoemulsions, composed of oil-in-water emulsions stabilized by cationic surfactants, facilitate mRNA encapsulation, cellular uptake, and endosomal escape, effectively triggering immune responses. Efforts continue to optimize these systems for better efficacy and safety in mRNA vaccine delivery (Zeng et al., 2023). However, due to issues with their biodegradability, dendrimers’ extensive clinical application is restricted. In the meanwhile, their branching polymer architectures provide the possibility of effective mRNA delivery with low toxicity (Karlsson et al., 2020).

The creation of vaccines, including those for COVID-19, has been one of the most well-known uses of mRNA technology. Because of its ability to be created quickly, mRNA is a perfect platform for reacting to pandemics. RNA treatments have potential not just for vaccinations but also for gene therapy and protein replacement therapies. Despite this promise, there are a number of obstacles facing mRNA-based therapies. These include of immunogenicity, instability, and challenges in effectively transporting mRNA to target cells. The use of mRNA in therapeutic applications is complicated by the fact that it might elicit immunological responses and is particularly vulnerable to destruction. Significant progress has been made in improving mRNA design, involving changes to the poly (A) tail, 5′cap, untranslated regions (UTRs), and coding sequences, in order to solve these problems. These modifications lengthen the time that proteins are expressed, increase translation efficiency, and stabilize mRNA. Furthermore, delivery vehicles like lipid nanoparticles (LNPs) are essential for shielding mRNA from deterioration and guaranteeing that it successfully reaches the target cells (Chavda et al., 2022; Al Fayez et al., 2023).

Self-amplifying RNA (saRNA), a more recent advancement in mRNA technology, is able to reproduce within cells. Lower amounts of mRNA can be employed to still produce the intended therapeutic effect since this replication process increases the creation of therapeutic proteins. Particular promise has been demonstrated by saRNA in the generation of vaccines, and it may find wider uses in therapeutic domains where persistent protein expression is necessary. To guarantee mRNA’s therapeutic effectiveness, production and purification are essential. The focus of the paper is on quality control while highlighting mRNA synthesis and purification techniques. Techniques such as high-performance liquid chromatography (HPLC) and RNase III treatment are required for eliminating contaminants, including double-stranded RNA, which can significantly influence the safety and efficacy of the mRNA product (Papukashvili et al., 2022).

The potential uses of mRNA delivery are many. In the field of protein therapy, hemophilia and other hereditary illnesses may now be treated using novel therapeutic proteins expressed via mRNA. These treatments are becoming more and more feasible because to developments in LNPs and ionizable lipids. Another exciting use is gene editing, where mRNA-based delivery of CRISPR-Cas9 technology is advantageous. This strategy lowers the dangers involved in long-term DNA integration, making gene editing a safer and more regulated process. The ability of this technology to be quickly deployed has been shown by the creation of mRNA vaccines, such as the COVID-19 vaccines, as mRNA may directly stimulate the body to produce antigens that trigger significant immune responses (Kowalski et al., 2019).

## 4 Customized mRNA vaccines: targeting for infectious pathogens and tumors

Customized mRNA vaccines have emerged as a transformative platform in modern medicine, offering unique advantages in targeting both infectious pathogens and cancer cells. Their mechanism of action revolves around introducing exogenous mRNA into somatic cells, where the mRNA is translated into specific antigens. These antigens are then recognized by the immune system, triggering both innate and adaptive immune responses. This capacity for highly specific immune activation has made mRNA vaccines a pivotal tool in immunotherapy and disease prevention, particularly in the context of cancer and rapidly mutating infectious diseases. Different from standard vaccine platforms, mRNA vaccines are developed quickly and can be highly customized (Li Y. et al., 2023). Conventional vaccines, including inactivated or live-attenuated vaccines, usually have long production schedules and are not as flexible in response to the tumor antigens’ or viral infections’ quick mutation. In contrast, mRNA vaccines offer unparalleled versatility as they may be developed and manufactured in a matter of weeks. This flexibility has come in very handy, particularly during the COVID-19 pandemic, when mRNA vaccines such as Moderna’s mRNA-1273 and Pfizer-BioNTech’s BNT162b2 were created and made available at a never-before-seen rate. These vaccinations proved to be highly effective and particularly targeted the SARS-CoV-2 spike protein, so demonstrating the viability of mRNA vaccine technology (Ankrah et al., 2023).

mRNA vaccines do not integrate into the host genome; therefore, there is no risk of associated genetic alterations. Regarding DNA vaccines, although the likelihood of integration into the host genome is very low, it is still considered a theoretical risk (Kozak and Hu, 2024). Furthermore, the mRNA is biodegradable, which means that after serving its purpose, the body breaks it down spontaneously and expels it. This temporary character reduces the possibility of long-term immune activation or off-target consequences, which are issues with conventional therapeutic approaches. These elements, along with the platform’s flexibility, make mRNA vaccines a potentially useful treatment for autoimmune disease, cancer immunotherapy, infectious diseases, and even a few uncommon genetic problems. The creation of efficient delivery mechanisms is essential to the success of mRNA vaccines because mRNA’s intrinsic instability poses a significant obstacle. Because nucleases in the bloodstream can quickly degrade mRNA, protective carriers are required. Because lipid nanoparticles (LNPs) can encapsulate mRNA, protect it from degradation, and promote its uptake into cells, they have become the most popular delivery vehicle. Additionally, LNPs improve mRNA’s biodistribution, making it more effective in reaching target cells. Beyond protection, LNPs increase the potential uses of mRNA-based therapeutics by enabling controlled release of mRNA and allowing it to be tailored to target particular tissues or cell types (Zhang et al., 2023a; Swetha et al., 2023).

With recent improvements in delivery efficiency and safety, LNPs are now the preferred delivery strategy for the majority of mRNA vaccine clinical applications. When mRNA vaccines are delivered to cells, they activate both the innate and adaptive immune systems, which results in a powerful immunological response. An essential part of the immune system, dendritic cells (DCs), process and deliver the encoded antigens to T cells, which is how they help with this process. This antigen presentation is crucial for the activation of cytotoxic T lymphocytes, which are responsible for recognizing and eliminating contaminated or malignant cells (Swetha et al., 2023). One characteristic of mRNA vaccines that contributes to their great efficiency is the simultaneous activation of innate and adaptive immune responses. Furthermore, pro-inflammatory cytokines generated in response to mRNA vaccines have the power to boost immunity and raise the effectiveness of the shot as a whole. This ability to stimulate distinct immune system branches provides comprehensive protection against the development of tumors and viral infections. The capacity of mRNA vaccines to alter the tumor microenvironment (TME) is a noteworthy therapeutic development in the field of cancer. Immunosuppressive microenvironments are frequently created by tumors to help them avoid immune surveillance (Verbeke et al., 2022). By encouraging immune cell infiltration into the tumor site, such as that of cytotoxic T lymphocytes (CTLs), improving antigen presentation, and stimulating an anti-tumor immune response, mRNA vaccines have the potential to upset this delicate equilibrium. One notable mechanism by which mRNA vaccines modulate the TME is through the polarization of macrophages. Tumor-associated macrophages (TAMs) are often skewed towards an M2 phenotype, which supports tumor growth and immune evasion. mRNA vaccines can shift TAM polarization towards the M1 phenotype, which has pro-inflammatory and anti-tumor properties. This reprogramming of the TME not only inhibits tumor growth but also enhances the effectiveness of other immunotherapies, such as checkpoint inhibitors (Huang et al., 2024; Table 2).

**TABLE 2:** Summary of mRNA-based therapeutic and preventive vaccine applications across various medical fields.

Application area	Target disease/Condition	Mechanism	Advantages	Challenges	Current research stage	Potential future applications
Infectious Diseases	COVID-19	Encodes spike protein of SARS-CoV-2 to induce immune response	Rapidly developed and high efficacy against variants	Variants may reduce vaccine effectiveness; public hesitancy	Approved and widely distributed	Updated vaccines for emerging variants (Khan et al., 2021)
	Influenza	mRNA encoding hemagglutinin to stimulate immunity	Faster production, can target multiple strains	Seasonal variation in strains can complicate efficacy	Ongoing clinical trials	Universal flu vaccine (Cheng et al., 2024)
	Zika Virus	Encodes Zika virus proteins for immune activation	Quick deployment during outbreaks	Limited outbreak zones may affect research scope	Phase 1 clinical trials	Other arboviral diseases (e.g., dengue) (Trovato et al., 2020)
	Rabies	Encodes rabies glycoprotein to provoke immunity	Reduced risk during production	Efficacy may be lower in certain populations	Clinical trials ongoing	Rabies prophylaxis in endemic regions (Fang et al., 2024)
	HIV	mRNA encoding HIV antigens to elicit immune response	Potential to stimulate broad immune activation	Complexity of HIV mutations; need for lifelong treatment	Early-stage research and trials	Preventative vaccines for other viral STIs (Fortner and Bucur, 2022)
Cancer Immunotherapy (Vishweshwaraiah and Dokholyan, 2022a)	Melanoma	Encodes tumor antigens to activate T cells against cancer	Personalized approach, targeted immune response	Variability in individual immune responses; potential for autoimmunity	mRNA-4157 (Moderna) in trials	Combination therapies with checkpoint inhibitors
	Lung Cancer	Encodes neoantigens specific to lung tumors	Precise targeting of tumor cells with minimal side effects	Tumor heterogeneity complicates treatment response	Ongoing clinical studies	Therapeutic vaccines for advanced stages
	Prostate Cancer	Encodes prostate-specific antigens for immune attack on tumors	Personalized therapy based on tumor profile	Variability in response; need for booster doses	Early clinical trials	Targeting other cancer types with similar profiles
	Breast Cancer	Targets HER2/neu protein to boost anti-tumor immunity	May enhance effectiveness of existing treatments	Potential side effects from immune activation	Preclinical studies in progress	Combining with hormone therapies for more effectiveness
Autoimmune Diseases (Deyhimfar et al., 2024)	Multiple Sclerosis (MS)	Encodes autoantigens to induce immune tolerance	Potential to reset immune tolerance without broad immunosuppression	Risk of exacerbating symptoms during treatment	Preclinical models	Targeting other neurodegenerative diseases
	Type 1 Diabetes	Encodes islet autoantigens to prevent beta cell destruction	Targeted immune modulation to delay onset.	Long-term efficacy and safety still under investigation	Preclinical development	Therapeutic approaches for other autoimmune diseases
	Rheumatoid Arthritis	Encodes joint-specific autoantigens to modulate immune response	Less systemic impact compared to traditional therapies	Difficulties in identifying the most effective antigens	Research in early phases	mRNA therapies for other inflammatory conditions
	Lupus	Encodes antigens associated with lupus for immune tolerance	Aims to reduce disease flare-ups	Complex pathophysiology complicates treatment outcomes	Preclinical exploration ongoing	Targeting other systemic autoimmune diseases
Genetic Disorders (Wadhwa et al., 2020)	Cystic Fibrosis	Delivers functional CFTR protein to correct chloride transport	Direct protein delivery without permanent gene alteration	Delivery efficiency and duration of effect may vary	Preclinical studies ongoing	Gene editing approaches combined with mRNA therapies
	Hemophilia	Encodes clotting factors (e.g., Factor VIII or IX) for bleeding episodes	Non-permanent treatment option, safer than viral vectors	Potential for immune responses against delivered proteins	Research in preclinical stages	Combined therapies for increased efficacy
	Duchenne Muscular Dystrophy	Delivers dystrophin mRNA to restore protein function in muscle cells	Potential for significant improvement in muscle function	Delivery to muscle tissue can be challenging	Ongoing clinical trials	Targeting other muscular dystrophies
	Phenylketonuria (PKU)	Encodes phenylalanine hydroxylase to restore enzyme function	Aims to reduce dietary restrictions	Long-term effectiveness and patient compliance	Early-stage trials	Broader metabolic disorders
Cardiovascular Diseases (Zhang et al., 2023a)	Atherosclerosis	Delivers proteins that reduce LDL cholesterol or promote plaque clearance	Targeted therapy with fewer systemic effects	Long-term impact and safety need further research	Early-stage research	Treatment strategies for other lipid disorders
	Heart Failure	mRNA encoding proteins to enhance cardiac function and repair	Potential to regenerate cardiac tissue post-injury	Variability in individual responses to therapy	Preclinical research ongoing	Gene delivery strategies for heart regeneration
	Hypertension	Targets pathways regulating blood pressure via mRNA delivery	Non-invasive approach to manage blood pressure	Identifying the most effective mRNA targets remains challenging	Research in early phases	mRNA therapies for other cardiovascular risk factors
Neurological Disorders (Yang et al., 2025)	Alzheimer’s Disease	Encodes amyloid-beta or tau proteins to reduce plaque formation	Early intervention potential with lower side effects	Complex mechanisms of neurodegeneration may complicate treatment	Preclinical studies underway	Preventative approaches for other neurodegenerative disorders
	Parkinson’s Disease	Encodes alpha-synuclein to slow neurodegeneration	Offers a novel approach to modifying disease progression	Need for lifelong treatment and monitoring	Research in preclinical stages	Targeting other synucleinopathies
	Multiple System Atrophy	Targets specific aggregates involved in the disease process	Potential for disease-modifying effects	Limited understanding of disease mechanisms may impede progress	Early-stage exploration	Therapies for other atypical parkinsonian syndromes

Abbreviations: STIs: Sexually Transmitted Infections, CFTR: cystic fibrosis transmembrane conductance regulator, LDL: Low-Density Lipoprotein.

Beyond cancer and infectious disorders, mRNA vaccines have a great deal of promise. Current studies are investigating their potential uses as platforms for regenerative medicine as well as in the treatment of genetic disorders and autoimmune illnesses. mRNA vaccinations may be developed to increase immunological tolerance to certain autoantigens in autoimmune disorders, a condition in which the body’s own tissues are erroneously attacked by the immune system (Li Y. et al., 2023). mRNA-based therapies can be employed to introduce functional copies of appropriate genes in hereditary diseases caused by protein deficiencies or malfunctions. The breadth of mRNA technology as a therapeutic platform is demonstrated by these possible uses. Despite mRNA vaccines’ enormous promise, a number of obstacles still need to be overcome. One of the main challenges is mRNA’s instability, which shortens its shelf life and necessitates strict storage guidelines like extremely low temperatures. This has been an enormous logistical challenge, especially in low-resource regions, during the worldwide COVID-19 vaccination distribution. To get over these obstacles and increase the availability of mRNA vaccines everywhere, advancements in mRNA formulation and stability are required. An further obstacle is the possibility of immunological-related adverse effects, such autoimmune or inflammation, which may arise from the intense immune activation brought on by mRNA vaccines. While such side effects have generally been mild in clinical trials, more research is needed to better understand and mitigate these risks, especially in vulnerable populations or for chronic therapeutic use (Qin et al., 2022; Table 2).

mRNA vaccine technology has shown promise in treating autoimmune diseases and genetic disorders. In autoimmune diseases, mRNA vaccines can be designed to induce immune tolerance by encoding specific autoantigens, thereby retraining the immune system to recognize self-proteins as non-threatening. For instance, studies have explored using mRNA to produce anti-idiotypic antibodies, offering a novel approach to prevent and treat autoimmune conditions (Niazi, 2023a).

In genetic disorders, mRNA therapies can provide instructions for producing functional proteins that are deficient or defective due to genetic mutations. Researchers are developing mRNA-based treatments that instruct cells to repair genetic defects, potentially curing diseases such as certain congenital blood disorders (Bara-Ledesma et al., 2025).

These advancements highlight the versatility of mRNA technology beyond infectious diseases, offering potential therapeutic strategies for a range of complex conditions.

The goal of recent advancements in mRNA transport and engineering has been to enhance mRNA vaccines’ safety profile and immunogenicity. Modified nucleosides have been included into the mRNA sequence as a method to lessen inflammatory reactions and the activation of innate immune sensors. Furthermore, novel materials and formulations that can improve tissue-specific targeting are being investigated as part of continuing advancements in nanoparticle design, which will increase the accuracy and effectiveness of mRNA vaccines. The therapeutic value of mRNA vaccines may be expanded to cover a wider spectrum of disorders if the mRNA sequence and delivery method are optimized (Maruggi et al., 2019). Finally, since they provide unmatched flexibility, safety, and efficacy, tailored mRNA vaccines have completely transformed the fields of immunotherapy and vaccination. Their quick production, customization to particular patients or pathogens, and strong immune activation make them a perfect platform for fighting cancer, infectious illnesses, and other ailments. But in order to properly utilize this technology, it will be necessary to solve the issues of mRNA instability, distribution optimization, and possible adverse effects. As science progresses, mRNA vaccines have the potential to be a major player in precision medicine’s future, offering ground-breaking answers to some of the most important medical problems of our day (Al Fayez et al., 2023).

## 5 New perspective in the development of mRNA vaccines

The advent of mRNA vaccines has marked a significant milestone in the field of immunology and vaccine development (Zhou et al., 2023). This innovative approach has not only revolutionized the way we combat infectious diseases but also opened new avenues for therapeutic interventions against various pathogens and even cancer (Miao et al., 2021; Kutikuppala et al., 2024). The development of mRNA vaccines, particularly during the COVID-19 pandemic, has demonstrated their potential to be rapidly designed and produced, offering a flexible and scalable platform for vaccine development (Kutikuppala et al., 2024; Amanpour, 2021). The investigation of pathogenetic factors (Buchynskyi et al., 2024a; Buchynskyi et al., 2024b), such as the role of the microbiome (Durack and Lynch, 2019; Petakh et al., 2023a; Petakh et al., 2023b; Petakh et al., 2022a; Pavlo et al., 2023; Petakh et al., 2022b), alongside the development of targeted preventive strategies, remains a critical focus in managing infectious diseases (Ellwanger et al., 2021; Garcia et al., 2022). Despite the extensive availability of therapeutic agents (Buchynskyi et al., 2023; Buchynskyi et al., 2024c; Kamyshnyi et al., 2023; Zumla et al., 2016), including repurposed medications (Halabitska et al., 2024a; Halabitska et al., 2024b; Petakh et al., 2024; Pfab et al., 2021), the advancement of mRNA vaccines remains an urgent scientific priority. As we look towards the future, several emerging perspectives highlight the transformative potential of mRNA vaccines beyond their current applications.

### 5.1 Personalized vaccination strategies

One of the most promising new perspectives is the potential for personalized mRNA vaccines (Al Fayez et al., 2023). By leveraging genomic information, mRNA vaccines can be tailored to the individual, offering personalized protection against diseases. This approach is particularly promising in oncology, where mRNA vaccines can be designed to target specific mutations present in a patient’s tumor, potentially improving therapeutic outcomes (Chehelgerdi and Chehelgerdi, 2023; Kenoosh et al., 2024). mRNA vaccines have emerged as a promising and versatile platform for cancer immunotherapy, offering high potency, specificity, and scalability, with recent advances in design, delivery—particularly through lipid nanoparticles (Vishweshwaraiah and Dokholyan, 2022b). Personalized mRNA vaccines utilize genomic information to create a vaccine that is specific to the individual’s genetic profile (Valdés-Fernández et al., 2021). This involves sequencing the patient’s genome to identify unique mutations or antigens that are present in their cells (Bagger et al., 2024; Chen et al., 2024). mRNA vaccines, which were rapidly developed during the COVID-19 pandemic, show great promise for cancer therapy, offering unique personalization options, enhanced stability through engineering techniques, and the ability to elicit both innate and adaptive immune responses, with several promising clinical trials underway (Sayour et al., 2024). For cancer patients, this means identifying tumor-specific neoantigens that can be targeted by the immune system (Okada et al., 2022). Gene fusion neoantigens, which are highly immunogenic and widely shared across cancer types, represent a promising target for cancer immunotherapies, offering potential for prognostic biomarkers and novel treatments, including tumor vaccines and immune checkpoint blockade therapies (Ellwanger et al., 2021). The mRNA vaccine is then designed to encode these neoantigens, prompting the body to produce the corresponding proteins and mount an immune response against the cancer cells (Ni, 2023). In oncology, personalized mRNA vaccines have shown significant promise (Li Y. et al., 2023). mRNA vaccines have rapidly advanced the biopharmaceutical industry, particularly through the development of COVID-19 vaccines, and show great promise for future vaccines against cancer and infectious diseases, despite ongoing challenges and evolving prospects in this field (Chakraborty et al., 2021). By targeting neoantigens that are unique to a patient’s tumor, these vaccines can stimulate a robust immune response that specifically targets cancer cells while sparing healthy tissue (Li X. et al., 2023). Over the past decade, immunotherapy has transformed cancer treatment through the approval of immune-checkpoint inhibitors and chimeric antigen receptor T-cell therapies, while personalized neoantigen-based vaccines have shown promising results in eliciting tumor-specific T-cell responses, suggesting a novel and individualized approach for future cancer therapies (Blass and Ott, 2021). This precision reduces the risk of off-target effects and enhances the efficacy of the treatment (Guo et al., 2023). Clinical trials have demonstrated the potential of personalized mRNA vaccines to induce tumor regression and improve patient outcomes (Yao et al., 2024b). One of the major challenges in cancer treatment is tumor heterogeneity, where different cells within the same tumor can have distinct genetic profiles (Janku, 2014). Personalized mRNA vaccines address this issue by targeting multiple neoantigens simultaneously, ensuring that a broader range of tumor cells are targeted (Fan et al., 2023; Eskandari et al., 2024). This approach increases the likelihood of a successful immune response and reduces the chances of tumor escape (Starzer et al., 2022).

Personalized mRNA vaccines signify a significant advancement in oncology, leveraging genomic data to customize vaccines according to the unique mutations present in an individual’s tumor, thereby enhancing therapeutic efficacy and eliciting strong immune responses (Liu et al., 2023). These vaccines focus on tumor-specific neoantigens and tackle issues such as tumor heterogeneity, with the goal of improving patient outcomes while reducing the risk of off-target effects (Tojjari et al., 2023). Despite the promising results observed in clinical trials regarding tumor regression, continued research is essential to address existing challenges and to further refine this innovative strategy in cancer treatment.

### 5.2 Expansion to non-infectious diseases

While mRNA vaccines have primarily been developed for infectious diseases, there is growing interest in their application to non-infectious diseases, such as cancer and autoimmune disorders (Gu et al., 2020; Yu et al., 2023). The ability to encode any protein of interest makes mRNA a versatile platform for inducing immune responses against a wide range of antigens (Qin et al., 2022). This could lead to breakthroughs in the treatment of diseases that currently lack effective vaccines (Tian et al., 2022). One of the most promising applications of mRNA vaccines in non-infectious diseases is in cancer immunotherapy (Liu et al., 2024). mRNA vaccines can be designed to encode tumor-specific antigens, which are then presented by the body’s cells to the immune system, stimulating a targeted immune response against cancer cells (Katopodi et al., 2024). Advancing personalized medicine in brain cancer hinges on innovative strategies, particularly mRNA vaccines, which demonstrate robust immune responses, rapid development, and cost-effectiveness, while ongoing clinical trials explore their efficacy in combination with other therapies, highlighting the importance of continued research and collaboration (Lin et al., 2023). This approach has shown potential in treating various types of cancer, including melanoma, prostate, and breast cancer (Gupta et al., 2022; Rastin et al., 2013).

mRNA vaccines are also being explored for the treatment of autoimmune disorders (Niazi, 2023b). These vaccines can be designed to induce immune tolerance to specific self-antigens, potentially reducing the autoimmune response that characterizes diseases such as type I diabetes, multiple sclerosis, and rheumatoid arthritis (Kim et al., 2023; Sharkey and Thomas, 2022). By encoding proteins that are involved in the autoimmune response, mRNA vaccines can help retrain the immune system to recognize these proteins as non-threatening, thereby alleviating the symptoms of the disease (Zhang et al., 2023b).

The potential of mRNA vaccines extends to chronic diseases, where they can be used to deliver therapeutic proteins that are deficient or dysfunctional in patients (Cooke and Youker, 2022; Kishore and Magadum, 2024). For example, mRNA vaccines can be engineered to produce insulin for diabetes patients or to replace defective proteins in genetic disorders (Elkhalifa et al., 2022; Makhijani et al., 2024b). Chemically modified mRNA presents a novel and efficient approach for developing biopharmaceutical agents, with proven effectiveness in COVID-19 vaccination and promising potential for treating various chronic diseases, including metabolic disorders, cancer, musculoskeletal, respiratory, cardiovascular, and liver conditions, facilitated by recent optimization advancements (Elkhalifa et al., 2022). This approach offers a novel way to manage chronic conditions by providing a continuous supply of therapeutic proteins directly within the patient’s body (Niazi and Magoola, 2023; Dingman and Balu-Iyer, 2019).

Research is underway to explore the application of these vaccines in non-infectious conditions, as they demonstrate potential for advancing personalized medicine through rapid development, cost-effectiveness, and efficacy in treating various cancers, while also fostering immune tolerance in autoimmune diseases (Abdelzaher et al., 2021; Szabó et al., 2022). These vaccines hold promise for the advancement of personalized medicine, exhibiting rapid development, cost-effectiveness, and potential efficacy in treating various cancers while also promoting immune tolerance in autoimmune diseases by retraining the immune system (Ramirez et al., 2024; Rabe et al., 2015). Furthermore, chemically modified mRNA presents an innovative strategy for managing chronic conditions by enabling the direct delivery of therapeutic proteins to patients, thereby enhancing the treatment of disorders such as diabetes and genetic diseases (Chabanovska et al., 2021; Petersen et al., 2024).

### 5.3 Rapid response to emerging pathogens

The COVID-19 pandemic demonstrated the speed at which mRNA vaccines can be developed and deployed (Chavda et al., 2022). This rapid response capability is a critical advantage in the face of emerging infectious diseases (Zhang et al., 2024). Future developments may focus on creating mRNA vaccine libraries for known pathogens, allowing for even faster deployment in outbreak situations (Zeng et al., 2022; Nitika and Hui, 2022).

The rapid response capability of mRNA vaccines represents a significant advancement in the field of vaccinology, particularly in the context of emerging pathogens (Ankrah et al., 2023; Maruggi et al., 2019). This capability is largely attributed to the unique properties of mRNA technology, which allow for swift design, production, and deployment of vaccines in response to new infectious threats (Mir and Mir, 2024; Yang et al., 2022).

One of the most critical advantages of mRNA vaccines is their rapid development timeline (Gote et al., 2023b). Traditional vaccine development can take years, often rendering them ineffective in the face of fast-spreading outbreaks (Jain et al., 2021). In contrast, mRNA vaccines can be designed and produced within weeks once the genetic sequence of a pathogen is known (Maruggi et al., 2019; Overmars et al., 2022). This was exemplified during the COVID-19 pandemic, where mRNA vaccines were among the first to be developed and authorized for emergency use (Park et al., 2021; Iqbal et al., 2024). This streamlined process eliminates the need for cultivating live pathogens, significantly reducing the time required to develop a vaccine (Excler et al., 2023; Elibe et al., 2023).

mRNA vaccines offer unparalleled flexibility and adaptability, which are crucial in responding to rapidly mutating pathogens (Wang Y. S. et al., 2023; González-Cueto et al., 2024). The modular nature of mRNA technology allows for quick updates to the vaccine formulation to address new variants of a virus (Wei et al., 2023). This adaptability was demonstrated with the development of updated COVID-19 vaccines targeting emerging variants (Zhao et al., 2024).

Moreover, mRNA vaccines can be designed to target multiple antigens simultaneously, providing broader protection against diverse strains of a pathogen (Kumari et al., 2024). This capability is particularly important for viruses like influenza and coronaviruses, which frequently undergo antigenic shifts and drifts (Yewdell, 2021; Wang et al., 2022).

The COVID-19 pandemic showcased mRNA vaccines’ critical advantage in addressing emerging infectious diseases (Kutikuppala et al., 2024; Hu et al., 2024). This technology allows for the swift design and production of vaccines (Ho et al., 2021). Additionally, the modular nature of mRNA vaccines facilitates quick adaptations to target multiple antigens and address emerging variants, providing broader protection against rapidly mutating pathogens (Chandra et al., 2024).

## 6 Discussion

mRNA vaccines have demonstrated versatility in both preventive and therapeutic applications, each leveraging distinct mechanisms to achieve their objectives. Preventive mRNA vaccines are designed to preemptively protect individuals from infectious diseases. They function by encoding the mRNA sequence of a pathogen-specific antigen, such as a viral spike protein. Upon administration, host cells translate this mRNA into the corresponding protein, which is then presented on the cell surface, prompting the immune system to recognize it as foreign. This process stimulates the production of neutralizing antibodies and primes immune memory cells, enabling a rapid and effective response if the actual pathogen is encountered in the future. A prominent example is the mRNA vaccines developed for COVID-19, which have been instrumental in controlling the pandemic.

In contrast, therapeutic mRNA vaccines aim to treat existing conditions, notably cancers and certain chronic infections. These vaccines encode tumor-associated antigens or neoantigens—proteins that are uniquely expressed or mutated in cancer cells. When introduced into the body, the mRNA directs the synthesis of these antigens, which are then displayed by antigen-presenting cells. This presentation activates CTLs that specifically target and destroy cells expressing the tumor antigens, thereby exerting an anti-tumor effect. Therapeutic mRNA vaccines can be personalized by identifying patient-specific tumor mutations, allowing for tailored treatments that enhance efficacy. Additionally, combining these vaccines with other therapies, such as immune checkpoint inhibitors, has shown promise in enhancing therapeutic outcomes.

Ensuring global accessibility of mRNA-based vaccines presents several challenges that must be addressed to achieve equitable immunization worldwide.

mRNA vaccines have emerged as a promising platform in cancer immunotherapy, offering high specificity, better efficacy, and fewer side effects compared to traditional treatments. These vaccines work by delivering mRNA sequences encoding tumor-associated antigens or neoantigens into the body, where they are translated into proteins that stimulate an immune response against cancer cells. One of the key advantages of mRNA vaccines is their exceptional safety profile. Unlike DNA-based therapies, mRNA vaccines do not integrate into the genome and are eventually degraded by normal cellular processes, presenting a lower risk of insertional mutagenesis. Additionally, mRNA vaccines can be rapidly designed and manufactured, allowing for swift responses to emerging cancer mutations or personalized treatment approaches.

Recent studies have demonstrated the potential of mRNA vaccines in treating various cancers. For instance, an investigational mRNA vaccine showed promise in patients with advanced-stage cancers, including lung cancer and melanoma, by inducing immune responses against the tumors. Additionally, a nanoparticle-based mRNA vaccine improved survival in dogs with brain cancer and induced rapid immune responses in a small study in humans with glioblastoma, indicating potential applicability across different cancer types.

In fact, mRNA vaccines represent a significant advancement in cancer immunotherapy, offering a flexible and efficient approach to elicit targeted anti-tumor immune responses. Ongoing research and clinical trials continue to explore their full potential in various cancer settings.

Cold Chain Requirements: mRNA vaccines, such as those developed for COVID-19, often necessitate stringent cold storage conditions to maintain stability. For instance, the Pfizer-BioNTech vaccine requires storage at ultra-low temperatures, which poses logistical challenges in regions lacking advanced refrigeration infrastructure. This limitation complicates distribution efforts, particularly in rural and low-income areas.

Manufacturing and Distribution Capacity: The production of mRNA vaccines involves complex processes, including the encapsulation of mRNA in lipid nanoparticles—a novel technology that has not been widely scaled before. This complexity can lead to bottlenecks in manufacturing and distribution, especially in low- and middle-income countries that may lack the necessary infrastructure and technological expertise.

Vaccine Hesitancy and Misinformation: Public skepticism towards mRNA vaccines, fueled by misinformation and concerns about safety and efficacy, poses a significant barrier to widespread adoption. Studies have identified widespread negative sentiment and a global lack of confidence in mRNA vaccines, underscoring the need for targeted communication strategies to foster acceptance and strengthen public trust.

Economic and Policy Barriers: High costs associated with mRNA vaccine production and distribution can make them unaffordable for many countries, creating disparities in vaccine access. Additionally, regulatory challenges and the need for international policy convergence complicate the global deployment of mRNA vaccines.

Addressing these challenges requires a multifaceted approach, including investment in cold chain infrastructure, expansion of manufacturing capabilities in underserved regions, effective public education campaigns to combat misinformation, and international collaboration to overcome economic and policy barriers.

## 7 Conclusion

In recent years, mRNA vaccines have emerged as a transformative approach in both infectious disease prevention and cancer immunotherapy. Their adaptability and rapid development capabilities have been instrumental in addressing various health challenges. In the realm of infectious diseases, mRNA vaccines have demonstrated high efficacy, as evidenced by their pivotal role in combating the COVID-19 pandemic. Beyond infectious diseases, mRNA vaccines are making significant strides in cancer treatment. By encoding tumor-associated antigens, these vaccines stimulate the immune system to recognize and attack cancer cells, offering a promising avenue for immunotherapy. Ongoing research and clinical trials continue to explore and expand the applications of mRNA vaccine platforms, underscoring their potential to revolutionize medical practice across various domains.

### 7.1 Definitions of new terms

#### 7.1.1 Neoepitope

A neoepitope is a novel peptide sequence resulting from tumor-specific mutations. These unique sequences are presented on the surface of cancer cells by MHC molecules and are recognized by the immune system as foreign, thereby eliciting a targeted immune response.

#### 7.1.2 Neoantigen

Neoantigens are a subset of antigens that arise from tumor-specific mutations, leading to the formation of new peptide sequences not present in normal tissues. These antigens are recognized by the immune system as non-self and can be targeted in cancer immunotherapy strategies.

#### 7.1.3 Placebo-controlled phase

In clinical trials, a placebo-controlled phase refers to a study segment where the effects of the investigational treatment are compared against a placebo—a substance with no therapeutic effect. This design helps determine the actual efficacy and safety of the treatment by minimizing bias.
